# Cracking of Rubber Plates

**Published:** 1867-06

**Authors:** A. D. Hollrook

**Affiliations:** Waukesha, Wis.


### CRACKING OF RUBBER PLATES.

BY A. D. HOLLROOK, WAUKESHA, WIS.

IN the January number of the DENTAL REGISTER, is an article on the above subject.

The writer, Dr. Jackson, has given *his remedy*, but of the *cause*, not a word.

We have had some little experience in this direction, having had two plates returned broken, from front, about one-half way back the length of the plate, in a straight line.

On examination, we soon discovered the cause, in the first returned, which applies with equal force to the second, we found that the front blocks in the upper plate were set too close to the lower teeth, striking the canine teeth on the bevel, thus spreading the plate at every occlusion of the jaw.

I see no necessity of multiplying words, only to say, "an ounce of prevention is better than a pound of cure;" and I *know* that one minute's time in applying the eye teeth to an emery wheel would have saved the extra labor of making over those plates.

In all mechanical operations, each and every part must be made to fit. A deviation of one-sixteenth of an inch in the matching of cogwheels will cause continual jaring and eventual failure. So with artificial teeth. Let not the front blocks be too closly on the lower teeth, neither must any of the teeth strike on the sliding scale, else failure will be the inevitable result. If the work does not fail immediately, it will be a source of continual annoyance in throwing off the plate so long as the Dentist fails to observe this one thing.

The breakage of plates depends upon two things:

1st.—Their faulty construction and imperfect adaptation; and, 2d.—The hard usage of the wearer.

The first to be obviated by far more care of the Dentist in making the denture, than is usually bestowed.

Time, care and skill to all necessary extent, should be employed in the construction of anything so intimately connected with the welfare and comfort of our patients as a set of teeth.

Hard usage by the patients, whether by carelessness, or extra use, will be somewhat modified, by imbuing their minds with true notions of the real value of a set of teeth.

